# A new dimension to conservative dentistry: Air abrasion

**DOI:** 10.4103/0972-0707.62632

**Published:** 2010

**Authors:** Vivek S Hegde, Roheet A Khatavkar

**Affiliations:** Department of Conservative Dentistry and Endodontics, M. A. Rangoonwala Dental College, Pune, India

**Keywords:** Air abrasion, aluminium oxide, conservative cavity preparation

## Abstract

Air abrasion dentistry has evolved over a period of time from a new concept of an alternative means of cavity preparation to an essential means of providing a truly conservative preparation for preservation of a maximal sound tooth structure. The development of bonded restorations in combination with air abrasion dentistry provides a truly minimal intervention dentistry. This article reviews the development of air abrasion, its clinical uses, and the essential accessories required for its use.

## INTRODUCTION

Minimally invasive dentistry is the need of the hour as the conventional ‘extension for prevention’ is being challenged and the more conservative forms of operative intervention are being recommended.[1] Procedures that aid in the removal of carious hard tissue, causing little or no damage to the adjacent sound tooth structures are fast being researched.

The study of the use of air abrasion technology for dental applications initiated by Dr Robert Black in the 1940's was successfully introduced in 1951 with the Airdent air abrasion unit (S.S. White). In spite of showing promising results, the concept did not gain popularity due to three major factors. Firstly, air abrasion was not able to prepare cavities with well-defined walls and margins, and the materials during that time (mostly amalgam and direct or indirect gold) demanded such preparations since the concept of bonding had not been introduced. Secondly, the introduction of the air turbine handpiece in the late 1950s made conventional cavity preparations less time consuming. Thirdly, as high-velocity suction had not been developed, evacuation of the powder was difficult.

Though the basic concept of the air abrasion device has remained the same, it has experienced a rebirth not due to changes in the device per se, but due to improvements in bonding, restorative materials, isolation, and high volume suction.[2,3] Air abrasion can be best described as a pseudo-mechanical, non-rotary method of cutting and removing dental hard tissue. The terms “micro air-abrasion” and “kinetic cavity preparation” have been used synonymously to describe air abrasion. Studies have shown that the bonding of enamel and dentin surfaces prepared with air abrasion is much better than that with conventional carbide burs or acid etching;[4–6] with the introduction of flowable and nano-filled composites it is easier to restore cavities which do not confer with the specifications of GV Black's concepts. Another major concern regarding the powder particles has also been averted due to the use of isolation in the form of rubber dam and high volume evacuation devices. Air abrasion also has the advantage of decreased noise and vibration as compared to conventional rotary instruments.[7]

## HOW DOES IT WORK?

Air abrasion for restoration preparation removes tooth structure using a stream of aluminium oxide particles generated from compressed air or bottled carbon dioxide or nitrogen gas. The abrasive particles strike the tooth with high velocity and remove small amounts of tooth structure. Efficiency of removal is relative to the hardness of the tissue or material being removed and the operating parameters of the air abrasion device.

A number of parameters such as the amount of air pressure, particle size, quantity of particles passing through the nozzle, nozzle diameter of the handpiece, angulation of nozzle of the handpiece, distance from object, and time of exposure to the object vary the quantity of tooth removal and depth of penetration.

- Generally, air pressures range from 40 to 160 psi. The recommended levels are at 100 psi for cutting and 80 psi for surface etching.
- The most common particle sizes are either 27 or 50 μm in diameter. The larger particles allow the clinician to work faster but will result in comparatively larger-sized cavity preparations than those with the 27 μm particles.
- Higher particle flow rate will allow more particles to abrade the working surface faster.
- The speed of the abrasive particles when they hit the tooth depends upon the gas pressure, nozzle diameter, particle size, and distance from the surface.
- Typical operating distances from the tooth range from 0.5 to 2 mm. Further distances produce a more diffuse stream that results in a diminished cutting ability.
- A number of variations in tip angulations and nozzle diameters are available. Smaller nozzle diameters can be used for areas that are difficult to access. The various tip angulations allow easy placement and orientation of the handpiece thus easing the strain off the operator's hands.

## WHAT ARE ITS USES/APPLICATIONS?

Specific indications for use of air abrasion include caries removal; removal of small existing restorations; preparation of tooth structure for cutting or etching for the placement of composites, porcelain and ceramics; and as an adjunct to the conventional handpiece bur.

Some of the situations where the air abrasion has particularly proved a boon include:

1. Removal of superficial enamel defects – these are much easier with the air abrasives since they result in removal of less tooth structure than the drill.[8]
2. Air abrasion is an excellent tool for detection of pit and fissure caries – when clinical, radiographic, and patient risk factors make pit and fissure caries suspect, air abrasion can be used to remove the organic debris and determine if caries is present. Use of burs for this procedure would remove far more sound enamel than the few micrometers removed with air abrasion.
  In the event of the operator not locating any carious lesions, the area can easily be sealed using a pit and fissure sealant.
  If caries is limited to enamel, then a sealant or flowable resin-based composite can be placed.
  If caries penetrates into dentin, then the preventive restoration can be used with a heavily filled resin in deep or wide areas subjected to forces of mastication. Sealant material may be used to cover non-carious pits and fissures.
  Additionally, caries detector dyes may also be used in conjunction with air abrasives to detect incipient lesion and treat them appropriately.
3. Air abrasion can also be used for the removal of pit and fissure surface stain on enamel before placement of a resin-based composite restoration or porcelain veneers [Figure 1].

**Figure 1:**
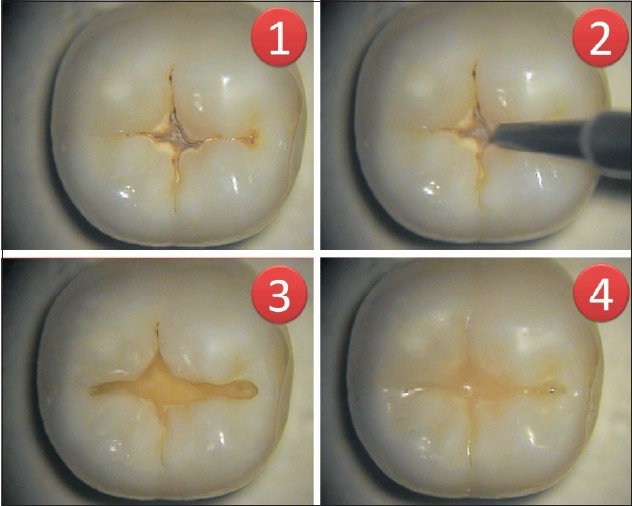
Air abrasion used to remove & restore pit & fissure caries using 27 micron-sized powder particles. 1) Fissure caries seen on occlusal surface of mandibular 2nd molar. 2) Tip of air abrasion device placed on molar. 3) Removal of caries with minimal cavity preparation width. 4) Cavity restored with preventive resin restoration. (Seen at 16X under dental operating microscope)
4. Teeth where the caries is restricted only to a small section of the tooth can also be prepared using air abrasives for conservation of sound tooth structure. Box-preparations for Class II cavities can also be prepared.
5. Surface preparation of abfractions and abrasions – air abrasion breaks the glaze of the highly polished surface that is not suitable for bonding and produces a highly textured surface that is excellent for the wet dentin-bonding technique.
6. Removal of existing restorations – the particles of the air abrasives can be used at higher pressures for removal of old amalgam restorations for replacing them or for removal and repair of composites, glass ionomers, and porcelain restorations [Figure 2].

**Figure 2:**
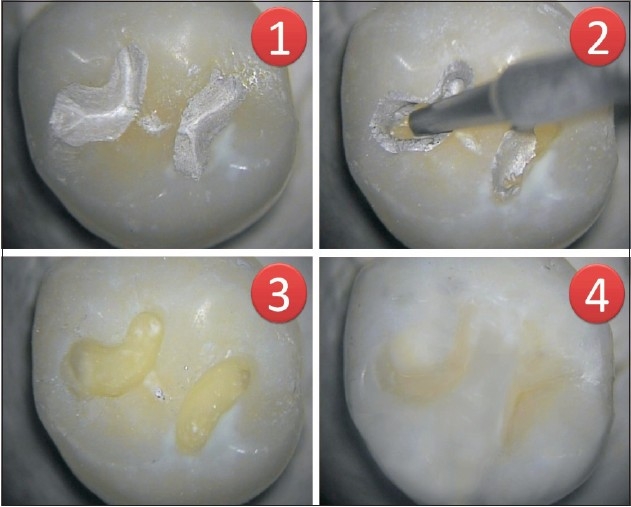
Air abrasion used to remove old amalgam restoration using 50 micron-sized powder particles followed by replacement with composite resins. 1) Amalgam restoration on maxillary molar. 2) Air abrasion device used to remove amalgam restoration. 3) Complete removal of amalgam restoration 4) Tooth restores with composite resin restoration. (Seen at 16X under dental operating microscope)
7. The use of local anesthesia while working in dentin may be avoided because of their cooling action through high pressure air.

### Method of clinical use

A number of air abrasion systems are available today such as the PrepMaster or EtchMaster (Groman Inc.), Airbrator (North Bay/Bioscience, LLC), PrepStart and PrepAir (Danville Engineering), or CrystalMark (CrystalMark Inc.) all of which work on the same principle. Some like the RONDOflex plus (KaVo) work on the principle of air abrasion technology with water spray. Operator controls are either mechanical or digital. Mechanical control is standard in most devices, and their control of powder flow rate (the primary determinant of overspray and consequent mess to be evacuated, washed or otherwise removed) is more tenuous than with digital control, which provides a consistent and minimal amount of powder while maintaining high efficiency. In selected devices digital control also allows for pulsed mode of operation, providing an interrupted air abrasive stream at settings from 0.5 to 2.0 seconds.

Air abrasion handpieces and nozzles are removable to facilitate sterilization and have working angles ranging from 0° to 120°. For precision cutting, as might be required for a preventive resin restoration, the 80° tip is more appropriate than the 45° tip. When shallow preparations are needed, as in the case of cervical erosion, the cutting patterns of the 45° tip are more appropriate.[9]

For facial and lingual preparations, a 60° angle produces a shallower preparation and allows for evacuation of reflected spray.

Nozzle orifice diameters range from 200 to 800 μm. Larger nozzle orifices require higher powder flow rates and gas pressures to maintain cutting efficiency.

Though the exposure to aluminum oxide particles may be considered a hazard, proper evacuation with high-volume suction takes care of it and can be supplemented with extra-oral evacuation systems that are designed to remove dry particles (but not any liquids). However, caution should be used with patients with known respiratory diseases, including asthma. Good suction and a rubber dam that extends over the nostrils will help to minimize inhalation of particles by the patient.

### Optional accessories for the air abrasion system

In addition to the different grades of the powder particles and the various tip diameter sizes and tip angulations for the air abrasion handpiece, there a few more accessories which will provide the clinician a better working environment:

1. Air abrasion resistant intraoral mirror: Majority of air abrasion operative dentistry procedures “eat up” an average of two to three mirrors per procedure, particularly when indirect vision is used. In an effort to conserve mirrors, the dentists will have a tendency to migrate towards direct vision, which in turn leads to obvious long-term deleterious effects on one's back. This mirror designed by CrystalMark Dental Systems, Inc. can withstand the indirect blasts of abrasive powder that are part and parcel of air abrasion dentistry. These mirrors come gold-plated for ease of identification by the staff and fit the standard no. 5 cone socket handle.
2. Sand trap: These are soft plastic spheres that slip onto office suction and have a top opening through which the air abrasive system tip is introduced. This device traps the abrasive particles within the sphere from where they can be evacuated through the suction. This prevents the abrasive particles from entering the patient's oral cavity [Figure 3].

**Figure 3:**
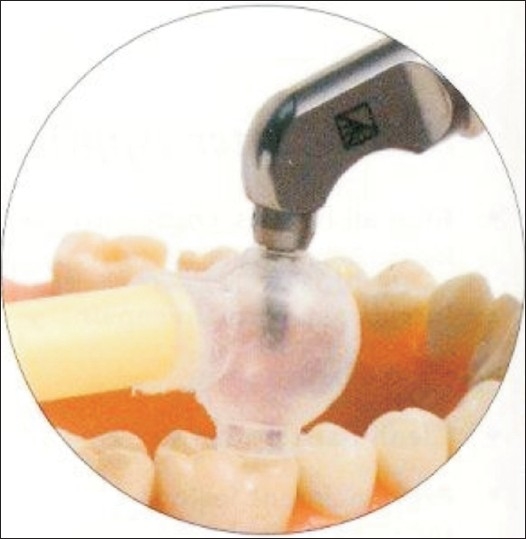
Sandtrap placed on mandibular molar demonstrating ease of debris evacuation
3. Power plus booster: Available as an accessory to the Prep Start (Danville Engineering) recompresses the compressed air up to 135 psi to increase the air pressure to allow for faster cutting thus reducing the patient chair-time.
4. Disposable air abrasion handpiece: The Airbrator^®^ (North Bay/Bioscience, LLC) is a single-use air abrasion handpiece that connects to your existing air-line. It is a direct alternative to traditional, expensive, self-contained air abrasion units. The Airbrator comes in three grades.
  High Performance – For small incipient lesions and cavity preparations.
  Medium Performance – For sealants, etching, bonding, and heavy stain applications.
  Light Performance – (Sodium Bicarbonate Powder) For removing stains, cleaning, and polishing.
  Others like the EtchMaster and PrepMaster^®^ (Groman Inc.) are pre-filled disposable air abrasion systems that can adapt to your handpiece connection for etching and intra-oral cavity preparations, respectively.
5. Super high volume evacuation systems: Like the RapidVac or Union Medical Evacuation System is the ideal companion for all air abrasive systems. Delivering super high volume suction, these devices completely eliminate the chances of contamination of the dental operatory with abrasive particles.
6. MicroVibe: Mechanical vibrations of the MicroVibe tip helps resin penetrate narrow gaps. It also improves the flow of pit and fissure sealants by increasing the contact between the sealant and tooth structure for effective restoration of cavities prepared using the air abrasives.

## DOES IT HAVE ANY LIMITATIONS?

Though air abrasives can be used in a large number of clinical situations there certainly are some limitations to their use such as

- Air abrasion is not an efficient means of removing large amalgam restorations especially, and there is concern for the levels of mercury released when amalgam is abraded. Air abrasion of amalgam for 1 min releases mercury vapor four times in excess of the OSHA standard. Same is true regarding removal of full coverage restorations.[10]
- Air abrasion is also not effective for removal of gross caries because it does not cut substances that are soft or resilient. In such cases, however, hand instruments like spoon excavators can be used to scoop out the soft lesions followed by air abrasives to remove the relatively hard carious tooth structures.
- Also the depth of penetration during cavity cannot be controlled, so it has to be accompanied with visual inspection in regular intervals.
- The splattering of the powder particles within the oral cavity and/or their accidental ingestion is another area of concern for which use of rubber dam isolation is a must. Additionally, patients, operator, and office staff must be equipped with protective eyewear to prevent the abrasive particles from accidentally entering the eyes.
- Air abrasive systems also cannot be used in conjunction with magnification devices such as loupes or dental operating microscopes as the rebound particles could cause damage to the lenses.
- Care must be taken when working near soft tissues due to risk of laceration, air dissection, and emboli. An inadvertent spray to soft tissues is not likely to cause damage, but a prolonged direct spray could potentially cause injury.[8,11]
- Air abrasion produces a rounded textured cavosurface margin and thus is not suitable for restorative preparations requiring definitive walls and sharp, well-defined cavosurface margins such as those needed for conventional amalgam and metal or porcelain inlays/onlays. It is also not suitable for crown preparations for either metal or porcelain coverage.[8]
- Though it has been claimed that air abrasion does not obviate the need for acid conditioning of enamel prior to sealant placement or composite resin restorations[12,13] there has been significant controversy regarding this issue, since a number of studies have also reported that a combination of air abrasion followed by acid etching has much better bond strengths compared to these procedures carried out individually.[14–16]

## CONCLUSION

The resurgence of air abrasive technology with newer restorative materials has given a new dimension to “minimally invasive dentistry.” The micro-mechanical bonding of the restoration to the tooth structure through maximum preservation of healthy tooth structure negates the need to follow conventional G.V. Black cavity design parameters. The dental profession finally has at its disposal as the modality with which to provide the patient with the ultimate in conservative dentistry. In addition to performing preventive procedures, the dentist also has the responsibility to educate the patients regarding the benefits of providing preventive dental care. A thorough knowledge, coupled with clinical experience will allow the clinician to use this new tool in most clinical situations.
